# MK2206 enhances the cytocidal effects of bufalin in multiple myeloma by inhibiting the AKT/mTOR pathway

**DOI:** 10.1038/cddis.2017.188

**Published:** 2017-05-11

**Authors:** Ru-Fang Xiang, Yan Wang, Nan Zhang, Wen-Bin Xu, Yang Cao, Jia Tong, Jun-min Li, Ying-Li Wu, Hua Yan

**Affiliations:** 1 Department of Hematology, Rui-Jin Hospital, Shanghai Jiao-Tong University School of Medicine, Shanghai 200025, China; 2 Department of Hematology, The Third Affiliated Hospital of Suzhou University, The First People’s Hospital of Changzhou, Changzhou 213003, Jiangsu Province, China; 3 Hongqiao International Institute of Medicine, Shanghai Tongren Hospital/Faculty of Basic Medicine, Chemical Biology Division of Shanghai Universities E-Institutes, Key Laboratory of Cell Differentiation and Apoptosis of the Chinese Ministry of Education, Shanghai Jiao Tong University School of Medicine, Shanghai 200025, China

## Abstract

Despite the development of promising cancer therapeutic drugs, multiple myeloma (MM) remains an incurable disease. Bufalin is a bufanolide steroid compound of the traditional Chinese medicine Chan Su that was previously shown to exert growth suppression effects on myeloma cell lines. Previous studies conducted by our group demonstrated that bufalin activated the AKT/mTOR pathway in myeloma cells, which is considered an essential pathway to disease progression and is related to drug resistance in MM. In view of the significant role of AKT in MM, the allosteric AKT inhibitor MK2206 was selected in order to enhance the antitumor effects of bufalin in different MM cell lines (NCI-H929, U266, LP-1 and RPMI8226). The data indicated that MK2206 enhanced the cytotoxicity of bufalin in MM cells, via the suppression of cellular proliferation and the induction of apoptosis, as demonstrated by cleavage of apoptosis-related proteins. This effect was further noted in the presence of exogenous interleukin-6 and/or following the co-culture of MM cells with bone marrow stromal cells (BMSC). This process was associated with the inhibition of the AKT/mTOR pathway. The combination of bufalin with MK2206 reduced the secretion of IL-6 in U266 cells. The combined treatment exhibited similar anti-MM effects in bortezomib-resistant cell lines (NCI-H929R, U266R). In addition to the *in vitro* cell line models, the synergistic effect was noted in primary MM cells and in MM xenografts of BALB-c and NOD-SCID mice. In conclusion, the data suggested that MK2206 significantly enhanced the cytocidal effects of bufalin in MM cells, regardless of the sensitivity to bortezomib, via the inhibition of the AKT/mTOR pathway. The study provided the basis of a promising treatment approach for MM.

Multiple myeloma is a heterogeneous hematological malignancy. It is estimated that 30 330 new cases and 12 650 associated deaths have been reported in the United States in 2016 due to MM.^1^ The use of new drugs namely, proteasome inhibitors (PIs), immunomodulatory drugs (IMiDs) and cell signaling protein inhibitors has contributed significant improvements in MM.^2^ Despite the promising advances in the development of MM therapy, MM remains incurable. Thus, more potent and selective drugs that target MM tumor cells are required in order to overcome drug resistance and improve patient outcome.

The AKT family of kinase enzymes is a key signaling partner of the PI3K pathway and consists of AKT1, AKT2 and AKT3. The latter enzymes play a pivotal role in cell survival and growth, and are frequently deregulated in a majority of human cancers.^3^ Previous studies have shown that the AKT kinase is activated in MM plasma cells, which sensitizes the anti-apoptotic pathway, mediates MM pathogenesis and accelerates disease progression.^4^ Furthermore, the activation of AKT is involved in osteoclast formation that can in turn cause osteolysis.^5^ On the basis of these studies, AKT targeting is considered a rational strategy for MM treatment.^6^ MK2206 is a potent, oral allosteric AKT inhibitor that enhances the antitumor efficacy of chemotherapeutic agents.^7, 8, 9^ MK2206 is well tolerated and exerts optimal safety profile, as demonstrated in the first-in-human clinical trial.^10^

Bufalin, an active ingredient of the traditional Chinese medicine Chan Su,^11, 12^ has been reported to have antitumor effect on various types of cancers, including leukemia,^13, 14, 15, 16^ breast,^17^ lung, liver, and pancreatic cancers.^18^ The previous study conducted by our group demonstrated that bufalin induced cellular apoptosis in MM cells,^19^ whereas a more recent study indicated that bufalin induced phosphorylation of AKT (p-AKT) in MM cell lines, which may counteract the cytotoxic effect of this compound and cause drug resistance, partially due to hyperphosphorylation of AKT.^20^

In the present study, the synergistic effects that were induced by the combination of bufalin and MK2206 were investigated in various myeloma cell lines (H929, U266, LP-1 and RPMI8226). A total of two out of four cell lines namely, H929R and U266R are bortezomib resistant. Furthermore, the combination treatment moderately enhanced the cytotoxicity and augmented apoptosis in myeloma cells via suppression of the AKT/mTOR pathway and the downregulation of Bcl-2 and Mcl-1 proteins. The aforementioned effects were further noted in the presence of exogenous interleukin-6 and/or in co-culture with bone marrow stromal cells. In addition, a synergistic effect of bufalin and MK2206 was observed in primary MM cells that was proportional to that noted in a xenograft mouse model. Thus, co-treatment of bufalin and MK2206 may be a promising strategy for the treatment of MM.

## Results

### MK2206 potentiated growth inhibition induced by bufalin in myeloma cells

**Figure 1:**
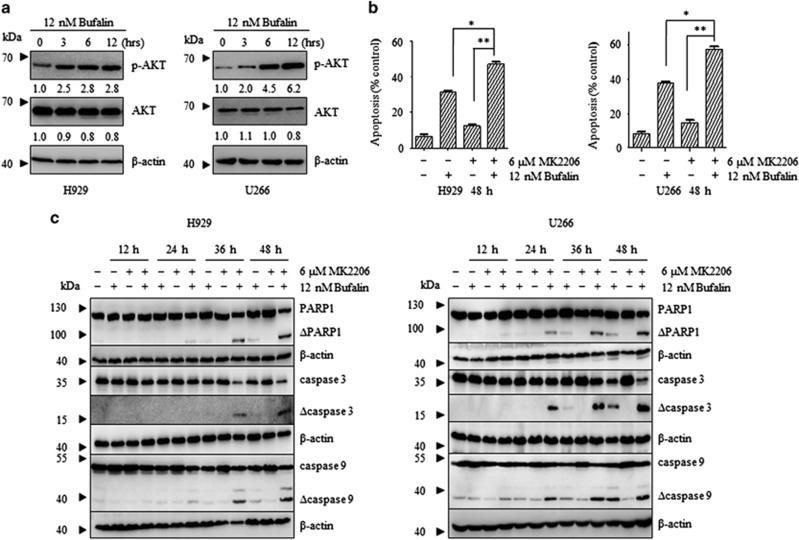
Bufalin-activated AKT in MM cells and the combination of bufalin with MK2206 increased the rate of apoptosis induction. (**a**) H929 and U266 cells were incubated with 12 nM of bufalin for 3, 6, 12 h and subsequently subjected to western blot analysis using anti-p-AKT, anti-AKT and anti-*β*-actin antibodies. *β*-actin was used as a loading control. (**b**) H929 and U266 cells were treated with 12 nM of bufalin and/or 6 *μ*M of MK2206 for 48 h and the apoptotic rates were analyzed by flow cytometry. The apoptotic effect of the combination group was statistically different compared with treatment of bufalin and/or MK2206 alone. Each bar represented the mean±S.E. of triplicate experiments. The values under the bands represented the mean quantitation ratios compared with the control groups. (**c**) H929 and U266 cells were treated with 12 nM of bufalin in the absence and/or presence of 6 *μ*M of MK2206 for 12, 24, 36, 48 h and the protein lysates were subjected to immunoblot analysis using antibodies specific against PARP, caspase-3, caspase-9 and/or *β*-actin. *β*-actin was used as a loading control. Experiments were performed in triplicate (*P<0.05; **P<0.01)

H929 and U266 myeloma cells were treated with 12 nM of bufalin for 48 h. The results indicated that bufalin alone moderately induced apoptosis in H929 and U266 cells (data not shown). The induction of apoptosis was accompanied with increased p-AKT levels and notably in U266 cells (Figure 1a). AKT plays a pivotal role in the development of myeloma due to the stimulation of cellular proliferation, the inhibition of apoptosis and the increase in myeloma cell motility.^21^ MK2206 is a potent allosteric AKT inhibitor that is currently evaluated in phase II trials for the treatment of solid tumors. Therefore, the effect of the combination of bufalin and MK2206 on cell viability was investigated in various types of MM cells, namely H929, U266, RPMI8226 and LP-1 cells. MK2206 potentiated the growth inhibition induced by bufalin in myeloma cells (*P*<0.05, Supplementary Figure S1A–D). CompuSyn synergism/antagonism analysis confirmed the synergistic effect of bufalin with MK2206 according to the CI value that was below 1.0 (Table 1). The results suggested that MK2206 enhanced the effect of bufalin in myeloma cells.

### The combined treatment of bufalin and MK2206 increased the induction of apoptosis via suppression of the AKT/mTOR pathway

The combination treatment of the two compounds on the induction of apoptosis was investigated in myeloma cells. H929 and U266 cells were exposed to 12 nM of bufalin alone and/or in combination with 6 *μ*M of MK2206 for 48 h (Figure 1b). The number of Annexin-V/PI^+^ cells increased in the combination treatment in H929 and U226 cells, respectively (*P*<0.05). The combination treatment resulted in increased levels of PARP cleavage and increased expression of cleaved caspases −3, −8 and −9 (Figure 1c and Supplementary Figure S2A) compared with the single treatment. A concomitant reduction in the expression of Bid was noted (Supplementary Figure S2A), although the levels of t-Bid (truncated Bid) were not investigated.

**Figure 2:**
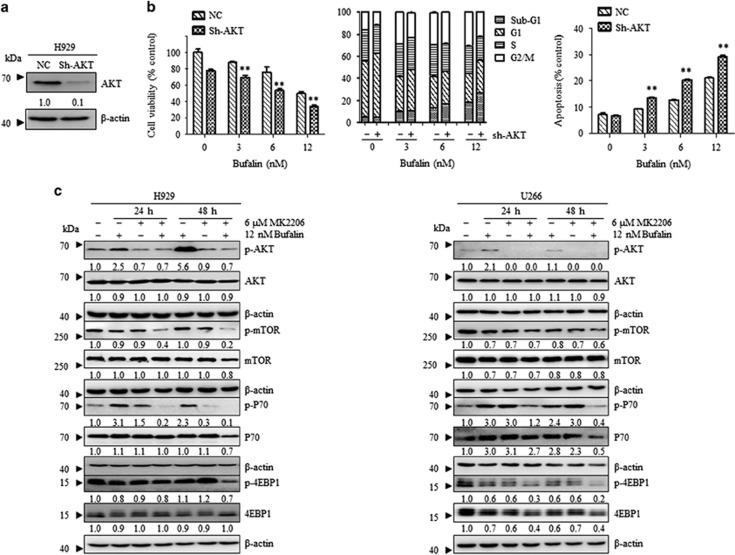
Induction of apoptosis following combination treatment of bufalin with MK2206 was associated with the inhibition of the AKT/mTOR pathway. (**a**) H929 cells were transfected with an AKT shRNA (H929 Sh-AKT) and/or a non-targeting shRNA (H929-NC) and protein lysates were subjected to Western blot analysis using antibodies specific against p-AKT and *β*-actin. (**b**) H929-NC and H929 Sh-AKT cells were incubated with 0, 3, 6, 12 nM of bufalin for 48 h. Cell viability was measured by CCK8 assay, whereas the cell cycle distribution was determined by flow cytometry analysis of the DNA content and cell apoptosis by the AnnexinV/PI assay. Each bar represented the mean±S.E. of triplicate experiments. (**c**) H929 and U266 cells were treated with 12 nM of bufalin in the absence and/or presence of 6 *μ*M of MK2206 for 24 and 48 h, and the levels of the phosphorylated and total AKT, mTOR, P70 and 4EBP1 proteins were examined by western blot analysis. *β*-actin was used as a loading control. The values under the bands were representative of the mean quantitation ratios compared with the control groups. Experiments were performed in triplicate (*P<0.05; **P<0.01)

In order to examine whether MK2206 increased the effect of bufalin via the inhibition of p-AKT, the expression of AKT was silenced in NCI-H929 cells using a sh-RNA sequence for AKT (shAKT-NCI-H929, Figure 2a). A decrease in cellular viability and cell cycle arrest with a concomitant induction of apoptosis were demonstrated in the presence of bufalin in shAKT-H929 cells compared with NC-H929 cells (Figure 2b). The combined effect of MK2206 and bufalin was consistent with the knockout of AKT in the presence of bufalin, indicating that the underlying mechanism may be associated with AKT inhibition. Furthermore, H929 and U266 cells were treated with 12 nM of bufalin alone and/or in combination with 6 *μ*M of MK2206. The data indicated that bufalin treatment alone increased the levels of p-AKT and its downstream signaling members namely, p-mTOR, p-P70S6K and p-4EBP1, whereas following addition of MK2206 the inhibition of the AKT/mTOR pathway was evident (Figure 2c). The data suggested that the addition of MK2206 enhanced the pro-apoptotic effect of bufalin via AKT inhibition.

### The induction of apoptosis by combined treatment of bufalin and MK2206 was partially related to the mitochondrial pathway

Previous studies have shown that cell death induced by inhibition of AKT is associated with the increase in the expression of the apoptotic proteins namely, Bcl-2 and Mcl-1. Bcl-2 and Mcl-1 belong to the anti-apoptotic members of the Bcl-2 family^22^ and are important regulators in the apoptotic pathway. The expression of Bcl-2 and Mcl-1 was significantly abolished following 48 h of combined treatment of bufalin and MK2206, notably in H929 cells (Supplementary Figure S3A). Consistent with this finding is the observation that the combination treatment resulted in a significant decrease of the mitochondrial potential (Supplementary Figure S3B).

### MK2206 enhanced apoptosis induction by bufalin in primary myeloma cells

**Figure 3:**
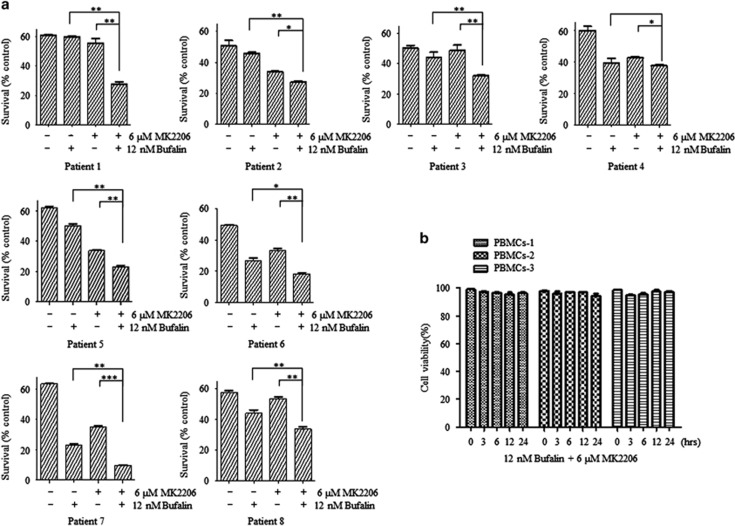
MK2206 enhanced the induction of apoptosis by bufalin in primary myeloma cells. (**a**) Patients’ mononuclear cells were separated by Ficoll–Hipaque density sedimentation and CD138-positive cells were isolated and treated with 12 nM of bufalin alone and/or in addition of 6 *μ*M of MK2206 for 48 h. The survival rates were assessed by Annexin V/PI staining. (**b**) Freshly isolated PBMCs from 3 healthy donors were cultured with 12 nM of bufalin and 6 *μ*M of MK2206 for 48 h. The viability was assessed by the tryphan blue assay. Each bar represented the mean±S.E. of triplicate experiments (*P<0.05; **P<0.01)

Mononuclear cells were isolated from bone marrow aspiration of 8 cases that presented with diagnosed MM (patient 1, 2, 5, 6, 7: IgG-*λ* subtype; patient 3: non-secretory myeloma; patient 4: IgA-*λ* subtype; patient 8: IgG-*κ* subtype). CD138^+^ myeloma cells were incubated with 12 nM of bufalin alone and/or in combination with 6 *μ*M of MK2206, and the induction of apoptosis was quantified. The data indicated that bufalin and MK2206 demonstrated a moderate ability to induce apoptosis, while the combination of bufalin and MK2206 demonstrated a substantial improvement in the induction of apoptosis in seven patients (except patient 4, *P*<0.05, Figure 3a). Bufalin alone or in combination with MK2206 exhibited no influence on cell viability in PBMCs obtained from healthy volunteers (Figure 3b).

### Bufalin and MK2206 abrogated the IL-6-mediated cell growth in myeloma cells and effectively reduced IL-6 secretion in U266 cells

**Figure 4:**
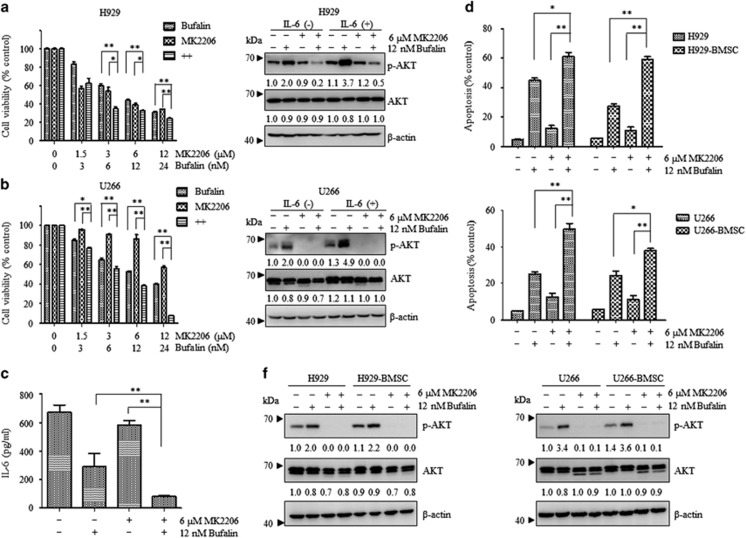
Bufalin and MK2206 surpassed IL-6-mediated cell growth and induced cooperative apoptosis of MM cells in the absence of BMSCs. (**a** and **b**) H929 and U266 cells were stimulated with IL-6 (10 ng/ml) for 1 h and subsequently cultured with the indicated reagents for 48 h. Cell viability was measured by the CCK8 assay. Cell lysates were subjected to western blot analysis using anti-p-AKT, anti-AKT, and *β*-actin Abs. *β*-actin was used as a loading control. The values under the bands were representative of the mean quantitation ratios compared with the control groups. (**c**) U266 cells were treated with 12 nM of bufalin in the absence and/or presence of 6 *μ*M of MK2206 for 48 h and subsequently centrifuged to obtain the supernatant. The samples were subjected to an IL-6 ELISA. (**d** and **f**) H929 and U266 cells were co-cultured with or without BMSCs for 48 h with 12 nM of bufalin in the presence and/or absence of 6 *μ*M of MK2206, and the apoptotic rates were measured by flow cytometry. Subsequently, the cell lysates were subjected to western blot using anti-p-AKT, anti-AKT and anti-*β*-actin Abs. *β*-actin was used as a loading control. The values under the bands were representative of the mean quantitation ratios compared with the control groups. Each bar represented the mean±S.E. of triplicate experiments (*P<0.05; **P<0.01)

IL-6 is a cytokine that is considered a significant survival factor in MM^23^ as demonstrated by *in vitro* and *in vivo* studies.^24^ The present study aimed to examine whether the drug combination could abrogate IL-6-mediated cell growth in myeloma. The growth of H929 cells was significantly inhibited following combination treatment of bufalin and MK2206 (Figure 4a). This effect occurred regardless of the presence of IL-6 (*P*<0.05) and was accompanied with an inhibition of p-AKT. In addition, a similar effect was observed in U266 cells (Figure 4b). The levels of IL-6 were tested under different treatment conditions, since U266 cells were shown to secrete this cytokine.^25^ Co-treatment of bufalin and MK2206 efficiently decreased IL-6 secretion, as demonstrated by ELISA (Figure 4c, *P*<0.01).

### Synergistic apoptotic effect was induced by bufalin and MK2206 during co-culture of MM cells and BMSCs

Previous studies have shown that cell–cell contact between MM cells and BMSCs plays a critical role in the survival and growth of myeloma cells. Adhesion of tumor cells to BMSCs activates a multitude of signaling pathways, leading to upregulation of cell cycle regulating and anti-apoptotic proteins.^26^ BMSCs were isolated from myeloma patients, and incubated in the presence of bufalin and MK2206 alone and/or in combination, in H929 and/or U266 cells, respectively. Bufalin and MK2206 moderately induced apoptosis of U266 and/or H929 cells in the presence of BMSCs. This effect was accompanied with a decrease of p-AKT and was independent of the contact of MM cells with BMSCs (Figure 4d–f). The results suggested that the combination of bufalin and MK2206 targeted MM cells directly and surpassed a cytoprotective effect that was mediated by the MM-host-BM microenvironment.

### The combination of bufalin with MK2206 overcame bortezomib resistance in bortezomib-resistant cells

**Figure 5:**
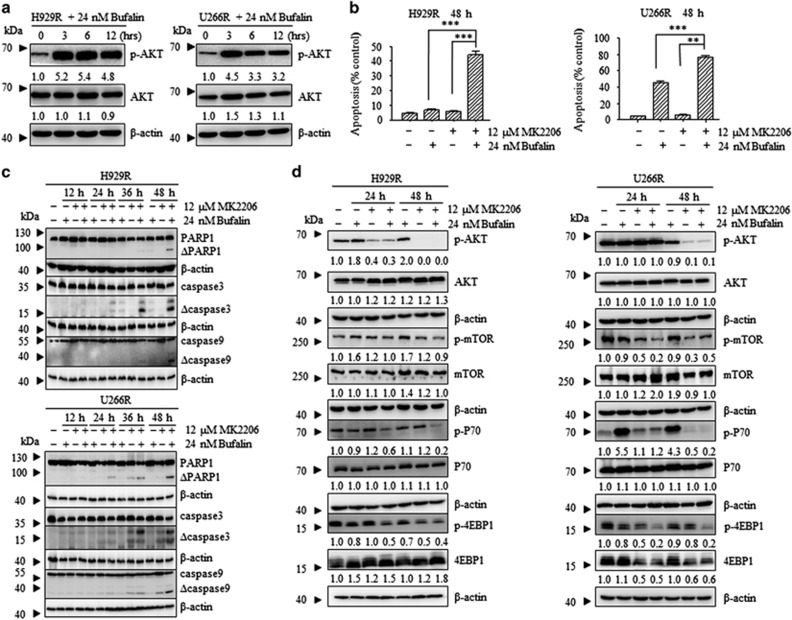
The combination of bufalin with Mk2206 surpassed bortezomib resistance in bortezomib-resistant cells. (**a**) H929R and U266R cells were treated with 24 nM of bufalin for 3, 6 and 12 h and subsequently subjected to Western blot analysis using anti-p-AKT, anti-AKT and anti-*β*-actin antibodies. *β*-actin was used as a loading control. The values under the bands were representative of the mean quantitation ratios compared with the control groups. (**b**) H929R and U266R cells were treated with 24 nM of bufalin and/or 12 *μ*M of MK2206 for 48 h, and the apoptotic rates were analyzed by Annexin V/PI assay. The combination group exhibited statistically different values compared with the treatment of bufalin and/or MK2206 alone. Each bar represented the mean±SE (standard error) of 3 independent experiments. (**c**) H929R and U266R cells were treated with 24 nM of bufalin in the absence and/or presence of 12 *μ*M of MK2206 for 12, 24, 36 and 48 h and protein lysates were subjected to immunoblot analysis using antibodies specific against PARP, caspase-3, caspase-9 and *β*-actin. *β*-actin was used as a loading control. (**d**) H929R and U266R cells were treated with 24 nM of bufalin in the absence and/or presence of 6 *μ*M of MK2206 for 24 and 48 h, and the levels of the phosphorylated and total AKT, mTOR, P70 and 4EBP1 proteins were examined by Western blot analysis. *β*-actin was used as a loading control. The values under the bands were representative of the mean quantitation ratios compared with the control groups (*P<0.05; **P<0.01)

In the present study, H929R and U266R cells were used in order to examine the cytocidal effect of bufalin and MK2206 in bortezomib-resistant myeloma cells (Supplementary Figure S4). Bufalin increased p-AKT levels in H929R and U266R cells (Figure 5a). Subsequently, H929R and U266R cells were treated with 24 nM of bufalin alone and 12 *μ*M of MK2206 and/or in combination for 48 h and subjected to apoptosis analysis. The combination treatment induced apoptosis in H929R and U226R cells, respectively (*P*<0.01, Figure 5b). The induction of apoptosis was accompanied with a decrease in Bid protein levels, PARP cleavage, and activation of caspases −3, −8, and −9 in a time-dependent manner (Figure 5c and Supplementary Figure S2B). Furthermore, the downstream signaling proteins of the AKT/mTOR pathway were inhibited in H929R and U266R cells (Figure 5d). Taken together, the findings suggested that the combination treatment of bufalin with MK2206 attenuated bortezomib resistance in bortizomib-resistant cells.

### Bufalin and MK2206 inhibited MM cell growth *in vivo*

**Figure 6:**
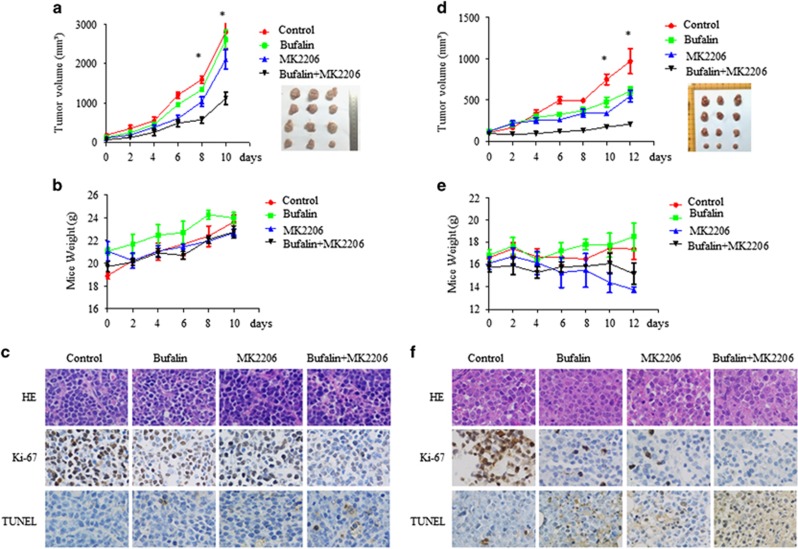
Bufalin and MK2206 inhibited MM cell growth *in vivo*. Mice bearing murine-derived MOPC315 MM tumors were treated with bufalin (1 mg/kg; intraperitoneally) daily in the presence and/or absence of MK2206 (120 mg/kg orally) for 10 days. Mice injected with human-derived H929 MM cells were treated with 1 mg/kg bufalin daily with or without 120 mg/kg MK2206 for 12 days. Tumor volume (**a** and **d**) and the weight of mice (**b** and **e**) were measured every 2 days. The tumor volume was calculated using the following formula: *V*=(length × width^2^)/2 (mean tumor volume±S.D., 3 mice per group). (**c** and **f**) Tumor sections from four groups were subjected to immunostaining using TUNEL and anti-Ki67 Abs that demonstrated tumor cell apoptosis and proliferation. Experiments were performed in triplicate (*P<0.05)

The efficacy of the combination treatment of the two regimens (bufalin and MK2206) was examined in a xenograft mouse model. Murine myeloma cells MOPC315 were injected in the right flanks of the BALB/c nu/nu female mice and when the tumor volume was in the range of 200 to 400 mm^3^, the mice were randomized into four groups. The groups were treated with vehicle or bufalin and/or MK2206 for 10 d and the mice were sacrificed in order to extract the tumors. The combined treatment significantly blocked MM tumor growth compared with the single treatment (Figure 6a, *P*<0.05). The mice did not exhibit significant side effects, such as weight loss, following bufalin and/or MK2206 treatment (Figure 6b). The combined treatment decreased tumor cell proliferation, as assessed by Ki67 staining, and increased the percentage of apoptotic cells compared to the vehicle, bufalin and/or MK2206 treatment as demonstrated by the increase of TUNEL-positive cells (Figure 6c).

The antitumor activity of the combination treatment was further assessed using a human MM (H929) xenograft model. In this model, H929 cells were injected subcutaneously in the right hind legs of NOD/SCID female mice and the treatment with vehicle, bufalin, MK2206 and/or combination was initiated when the tumor volume was in the range of 200 to 400 mm^3^. Following 12 days of treatment, NOD/SCID mice were killed and the tumor tissues were removed. Administration of bufalin and MK2206 resulted in a significant decrease in tumor volume compared with vehicle and/or single agent-treated animals (Figure 6d, *P*<0.05). This indicated that the combined treatment significantly inhibited MM tumor proliferation *in vivo* compared with the single treatment. Analysis of mouse weight revealed no significant differences between the treatment groups (Figure 6e). In addition, immunohistochemical analysis of Ki67 and TUNEL demonstrated inhibition of tumor cell proliferation and increased apoptosis in the tumors of the combined treatment group compared to the remaining three groups (Figure 6f).

## Discussion

Multiple myeloma is an incurable plasma cell malignancy characterized by a high rate of disease recurrence and drug resistance, which has stimulated the development of novel therapeutics in order to improve the patient outcome. Bufalin is a bufadienolide extract from the traditional Chinese medicine Chan Su,^27^ which has been widely used in China as an anodyne, cardiotonic, antimicrobial, local anesthetic and as a antineoplastic agent. Recent studies reveal that bufalin stimulates reactive oxygen species and inhibits the NF-*κ*B, STAT3 and AKT signaling pathways. The modulation of these pathways contributes to the antitumor effects of bufalin. Nevertheless, recent findings reported by our group indicated that bufalin induced phosphorylation of AKT (p-AKT) in myeloma cells. The underlying mechanism of this discrepancy is currently unknown. However, the difference may be attributed to the different cell types and cellular content of the tissues. Considering the prosurvival effect of AKT, we hypothesized that the activation of AKT may neutralize the antitumor effects of bufalin. In order to test this hypothesis, evidence was provided that inhibition of AKT can enhance the anti-MM effects of bufalin. Initially, it was demonstrated that the combination of bufalin with the novel small-molecule allosteric inhibitor of AKT namely, MK2206, exerted greater inhibitory efficacy in H929, U266, RPMI8226 and LP-1 cells, compared with single administration of the compounds. Similarly to the effect caused by the AKT inhibitor, the knockdown of AKT in MM cells further enhanced the inhibition of cellular proliferation and the induction of cellular apoptosis by bufalin.

IL-6 is the most significant growth factor with regard to the extended survival and drug resistance of MM. A previous study indicated that IL-6, could upregulate Mcl-1 expression, while the corresponding *Mcl-1* gene was downregulated solely following IL-6 starvation.^28^ Thus, the combination effects of the two treatments in myeloma cells following IL-6 stimulation were examined, and it was demonstrated that concomitant treatment of bufalin with MK2206 could successfully abrogate IL-6-mediated cell growth and reduce IL-6 secretion in U266 cells.

MM pathogenesis and progression is associated with the bone marrow (BM) microenvironment and BMSCs are considered the major cell types that comprise the BM. *In vitro* studies have suggested that cell–cell contact between MM cells and BMSCs exerts a significant role in the support of the survival and growth of the plasma cells.^29^ In order to further investigate whether bufalin and MK2206 induced apoptosis of myeloma cells in the presence of BMSCs, MM cells were co-cultured with BMSCs directly in the presence of bufalin and MK2206 alone and/or in combination. The results indicated that bufalin and MK2206 targeted MM cells directly and surpassed the cytoprotective effects mediated by the MM-host-BM microenvironment.

Adaptive resistance of myeloma to proteasome inhibitors represents a clinical challenge. Bortezomib is a first-class, reversible, boronate-type proteasome inhibitor widely used in MM.^30^ In order to determine whether the combination of bufalin with MK2206 overcomes bortezomib resistance in MM cells, the previously characterized bortezomib-resistant MM cell lines (H929R and U266R) were used. Bufalin indicated significant anti-MM activity in H929R and U266R cells, while the addition of MK2206 confirmed the efficacy of the combination treatment to overcome bortezomib resistance.

The synergistic effect of the two treatments in primary MM cells was confirmed by analysis of the samples derived from eight newly diagnosed MM patients. In addition, the combination treatment did not exhibit toxic effects on peripheral mononuclear cells derived from three healthy volunteers. Furthermore, the synergism between bufalin and MK2206 was confirmed by the MM xenograft mouse model, using in human-derived and/or murine-derived MM cells. Taken collectively, the data suggest that bufalin and MK2206 may be promising candidates that can be further studied in clinical trials of MM patients.

Although the treatment strategies of MM have significantly improved, the development of drug resistance remains a serious disadvantage of the clinical efficacy of the drugs used for this disease. The clonal evolution of myeloma cells, the changes in the bone marrow microenvironment, the deregulation of microRNAs and the signaling interaction with the programmed death factor 1 (PD-1)/PDL1 contribute to the drug resistance noted in MM.^31^ The induction of the AKT/mTOR signaling by cytokines in the BM microenvironment mediates resistance to conventional and novel therapies. Neither bortezomib nor IMiDs could block the AKT/mTOR pathway.^32^

Since MK2206 is an AKT inhibitor, and the effects of the combination treatment of MK2206 and bufalin were consistent with the knockout of AKT in the presence of bufalin. Previous studies reported that AKT and mTOR exhibit a complex interaction that is mediated by the modulation of PTEN and TSC1/2 protein expression.^33^ The mTOR kinase comprises two complexes namely, mTORC1 and mTORC2. The mTORC1 is regulated by P70S6K and 4EBP1 and the mTORC2 is regulated by AKT by phosphorylation of serine 473. The extended use of rapamycin and its derivatives that target mTORC1, inevitably leads to the development of tumor-drug-resistance due to the elevated AKT activity.^34^ MK2206 was documented to inhibit all members of the AKT/mTOR pathway by previous reports. In the current study a decrease in the phosphorylation of AKT, mTOR, P70 and 4EBP1 proteins was observed following the combination treatment that contributed to avoiding drug resistance, with regard to the AKT/mTOR signaling pathway.

## Materials and Methods

### Cell culture

The human MM cell lines NCI-H929 (referred to as H929), U266, LP-1, RPMI8226 and the murine MM cell line MOPC315 were purchased from the American Type Culture Collection (ATCC). H929R and U266R cells were established by constant stimulation using bortezomib at a gradually increasing concentration range. The cells were split twice every week and the bortezomib dose was gradually increased with administration of 4 nM of the compound per month to a final dose of 50 nM following 12 months of treatment. The cells were maintained in 50 nM of bortezomib for 3 months and subsequently in bortezomib-free medium for 2 weeks prior to the experimental treatment. All cell lines were cultured in RPMI-1640 medium (Sigma-Aldrich, St. Louis, MO, USA), supplemented with 10% (v/v) heat-inactivated fetal bovine serum (HyClone, Logan, UT, USA), 100 U/ml penicillin and 100 *μ*g/ml streptomycin (Invitrogen, Paisley, Scotland, UK), in a humidified incubator at 37 °C in the presence of 5% CO_2_/95% air.

### Reagents

Bufalin (3beta, 5beta-3, 14-Dihydroxy-bufa-20, 22-dienolide) was purchased from Tauto Biotech Co., Ltd., Shanghai, China, with >98% purity as determined by HPLC analysis. MK2206 and Captisol were from Selleck Chemicals. The aforementioned drugs were prepared in Dimethyl sulfoxide (DMSO), aliquoted and stored at −20 °C.

### Primary MM cells and normal mononuclear cells

Written informed consent was obtained from eight myeloma patients and three healthy volunteers prior to sample collection, for the use of their specimens in the study protocol. The study was conducted in accordance with the Declaration of Helsinki that ensures the safety and well-being of the subjects and the integrity of the data. The protocol was approved by the Clinical Investigational Reviewing Board of the Shanghai Second Medical University, China. Mononuclear cells (MNCs) were isolated from BM specimens of MM patients by Ficoll–Hipaque (Pharmacia, Piscataway, NJ, USA) density sedimentation. CD138^+^ cells were selected from MNCs using EasyStep CD138^+^ magnetic nanoparticles, as described in the instructions from the manufacturer’s protocol (Stem Cell Technologies, Vancouver, BC, Canada). Peripheral blood mononuclear cells (PBMCs) were obtained from healthy volunteers in the same way.

### Cell viability assay

MM cells (0.2–1 × 10^5^ per 200 *μ*l) were seeded in 96-well plates and incubated with various drug concentrations in triplicate for the indicated time periods. The cells were incubated with 10 *μ*l of Cell Counting kit-8 (Dojindo, Kumamoto, Japan), according to the instruction provided in the protocol of the manufacturer. The experiments were conducted three times.

### Cell cycle assay

Cells were treated with bufalin (0, 3, 6 and 12 nM) for 48 h and then collected and washed twice with phosphate-buffered saline (PBS). After fixed with 75% cold ethanol at −20 °C overnight, cells were incubated with RNase (100 mg/ml) for 30 min at 37 °C. Cells were stained with propidium iodide (Sigma-Aldrich, St Louis, MO, USA; 250 mg/ml) and incubated for another 15 min in the dark. Then, cells were analyzed by flow cytometry.

### Cell apoptosis assays

Quantification of apoptosis was determined by the Annexin-V apoptosis detection kit (BD Pharmingen) following the instructions of the manufacturer protocol. Annexin-V-positive and propidium iodide-negative cells were considered to be in the early apoptotic phase, whereas the cells that exerted positive staining for Annexin-V and propidium iodide were considered to be in the late apoptotic and/or necrotic stage. All data were collected, stored, and analyzed by LYSIS II software (BD Biosciences, San Diego, CA, USA). For primary myeloma samples, cells were selected from MNCs using EasyStep CD138+ magnetic nanoparticles and stained with Annexin-V-FITC, and propidium iodide at room temperature for 30 min. The cells were washed with 1 × binding buffer twice, and analyzed using a LSR II flow cytometer (BD biosciences).

### Western blot

MM cells were harvested, washed with PBS twice and lysed with lysis buffer (62.5 mM Tris-HCL, pH=6.8, 100 mM DTT, 2% SDS, 10% glycerol). The samples were centrifuged at 12 000 × *g* for 10 min. The protein concentration of the cell lysates was measured using the Bradford assay. The protein samples (10–40 *μ*g) were equally loaded on 6–15% SDS-polyacrylamide gels, electrophoresed and transferred to nitrocellulose membranes (Amersham Bioscience, Buckinghamshire, UK). Following 2 h of blocking with 5% of nonfat milk in PBS at room temperature, the membranes were incubated with different antibodies namely, anti-phospho (p)-AKT (Ser473), anti-AKT, anti-p-mTOR (Ser248), anti-mTOR, anti-ribosomal P70S6 kinase (P70S6k), anti-p-P70S6K (Thr389), anti-eukaryotic binding proteins (4EBP1), anti-p-4EBP1 (Thr70), anti-caspase-3, anti-cleaved caspase-3, anti-caspase-8, anti-Bid (Cell Signaling Technology, Beverly, MA, USA), anti-PARP1, anti-caspase-9 and anti-cleaved caspase-9 (Santa Cruz Biotechnology, CA, USA), overnight at 4 °C. The following morning the membranes were incubated with HRP-linked secondary antibodies for 2 h at room temperature. The protein bands were detected by chemiluminescence phototope-HRP kit (Merck Millipore, Jaffrey, NH, USA) according to the instructions provided by the manufacturer. HRP-linked anti-*β*-actin antibody was used as a loading control.

### Cytokine ELISA

U266 cells were plated at a density of 5 × 10^5^ cells per well and cell culture supernatants were collected following 48 h. The concentration of IL-6 was determined by ELISA assay kits (R&D Systems, Minneapolis, MN, USA), whereas the absorbance was measured using an ELISA plate reader at specific wavelengths determined by the assay kit.

### RNA interference

A retrovirus with target sites in the AKT gene that were previously reported^19, 35^ were packaged with plasmids containing a non-target control shRNA (NC) in HEK293T cells by co-transfection with pSIREN-RetroQ, pEQPAM (containing gag-pol, produced by Dr. Lishan Su at UNC, Chapel Hill, NC, USA) and VSVG (Clontech Laboratories, Mountain View, CA, USA, T-334350). Following transfection for 48 h, the viral supernatant was collected, filter-sterilized and added to H929 cells (2 × 10^5^ cells/well) in 6-well plates in the presence of a medium containing 8 *μ*g/ml of polybrene (Millipore, TR-1003-G). Puromycin (2.0 *μ*g/ml, Calbiochem, 540411) was used to select the stable-transfected cells at a 96 h time period.

### Co-culture

Bone marrow stromal cells (BMSCs) were isolated from bone marrow aspirates of MM patients as previously described.^26^ For direct co-culture, 5 × 10^5^ BMSCs cells were seeded in a 10 cm Petri dish and 5 × 10^5^ MM cells were added 24 h following BMSCs seeding. Following 48 h of incubation, the MM cell lines were collected.

### Xenograft mouse model

BALB-c nu/nu female mice aged 4–6 weeks and NOD-SCID female mice aged 4–6 weeks were used in the experiments. MOPC315 cells were injected subcutaneously in the right flanks of the BALB-c nu/nu female mice (0.2 ml per mouse containing 2 × 10^7^ cells) and the H929 cells (0.2 ml cell suspension containing 50% Matrigel (Corning, Bedford, MA, USA) per mouse and 1 × 10^7^ cells) were injected subcutaneously in the right hind leg of sublethally irradiated (200 cGy) female non-obese diabetes/severe combined immunodeficiency (NOD/SCID) mice. Tumor growth and mouse weight were monitored every 2 days. The mice were randomized into four groups (3 mice per group: vehicle control, bufalin, MK2206, bufalin+MK2206) when the tumor volume reached a range of 200 to 400 mm^3^. Bufalin was administered to the mice intraperitoneally at a daily dose of 1 mg/kg. MK2206 was administered to the mice orally, at a dose of 120 mg/kg, three times per week. Following successful treatment for 10 days, the groups of BALB-c nu/nu female mice were killed and the tumor tissues were removed. Following treatment for 12 days, the groups of NOD-SCID female mice were killed and the tumor tissues were removed. The difference in the time span of administration was due to the different cell types. The study was approved by the Shanghai Jiao Tong University School of Medicine Institutional Animal Care & Use Committee.^36^

### Statistical analysis

All experiments were carried out in triplicate. Statistical significance of differences observed in drug-treated versus control cultures was determined using the Wilcoxon signed ranks test. In all cases, a *P-*value of <0.05 (*P*<0.05) was considered statistically significant. The interaction between Bufalin and MK2206 was analyzed by isobologram analysis using the CompuSyn software program (ComboSyn, Inc., Paramus, NJ, USA) in order to determine whether the combination was antagonistic or synergistic. The combination index (CI) lower that was <1.0 indicated a synergistic effect, as previously described.

## Figures and Tables

**Table 1:** CI values of bufalin and MK2206 in MM cells

**Cell line**	**Bufalin (nM)**	**MK2206 (*****μ*****M)**	**Combination index**
NCI-H929	1.5	0.75	0.63151±0.17898
	3	1.5	0.35813±0.16165
	6	3	0.62726±0.15774
	12	6	0.39784±0.14055
	24	12	0.68673±0.11926
U266	1.5	0.75	0.74681±0.12682
	3	1.5	0.78902±0.05403
	6	3	0.63393±0.06415
	12	6	0.48425±0.05617
	24	12	0.54466±0.13783
RPMI8226	1.5	0.75	0.98908±0.23793
	3	1.5	0.54602±0.09387
	6	3	0.61654±0.11481
	12	6	0.69458±0.03525
	24	12	0.26182±0.08178
LP-1	1.5	0.75	0.97739±0.25743
	3	1.5	0.57839±0.12488
	6	3	0.57851±0.08621
	12	6	0.60430±0.05833
	24	12	0.54849±0.20227
H929R	6	3	0.20565±0.08265
	12	6	0.28958±0.06326
	24	12	0.46241±0.20279
U266R	6	3	0.20333±0.09171
	12	6	0.33709±0.09838
	24	12	0.59512±0.23565
