# Supplementary figures and images for: MK2206 enhances the cytocidal effects of bufalin in multiple myeloma by inhibiting the AKT/mTOR pathway
Source: Cell Death Dis. 2017 May 11;8(5):e2776–. doi: 10.1038/cddis.2017.188 (PMC5520709; doi:10.1038/cddis.2017.188)

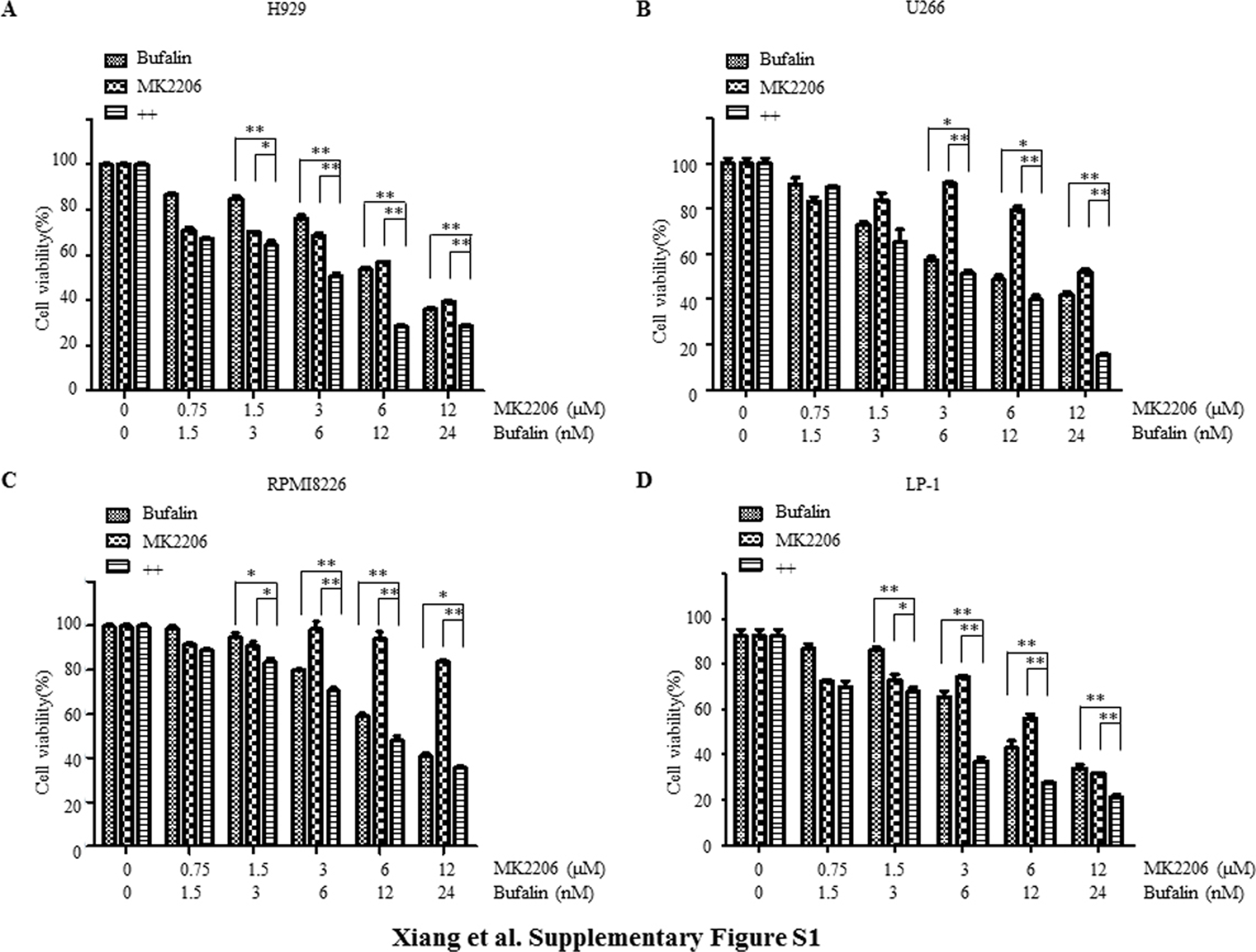

Supplement: Supplementary Figure S1 [file cddis2017188x1.tif]

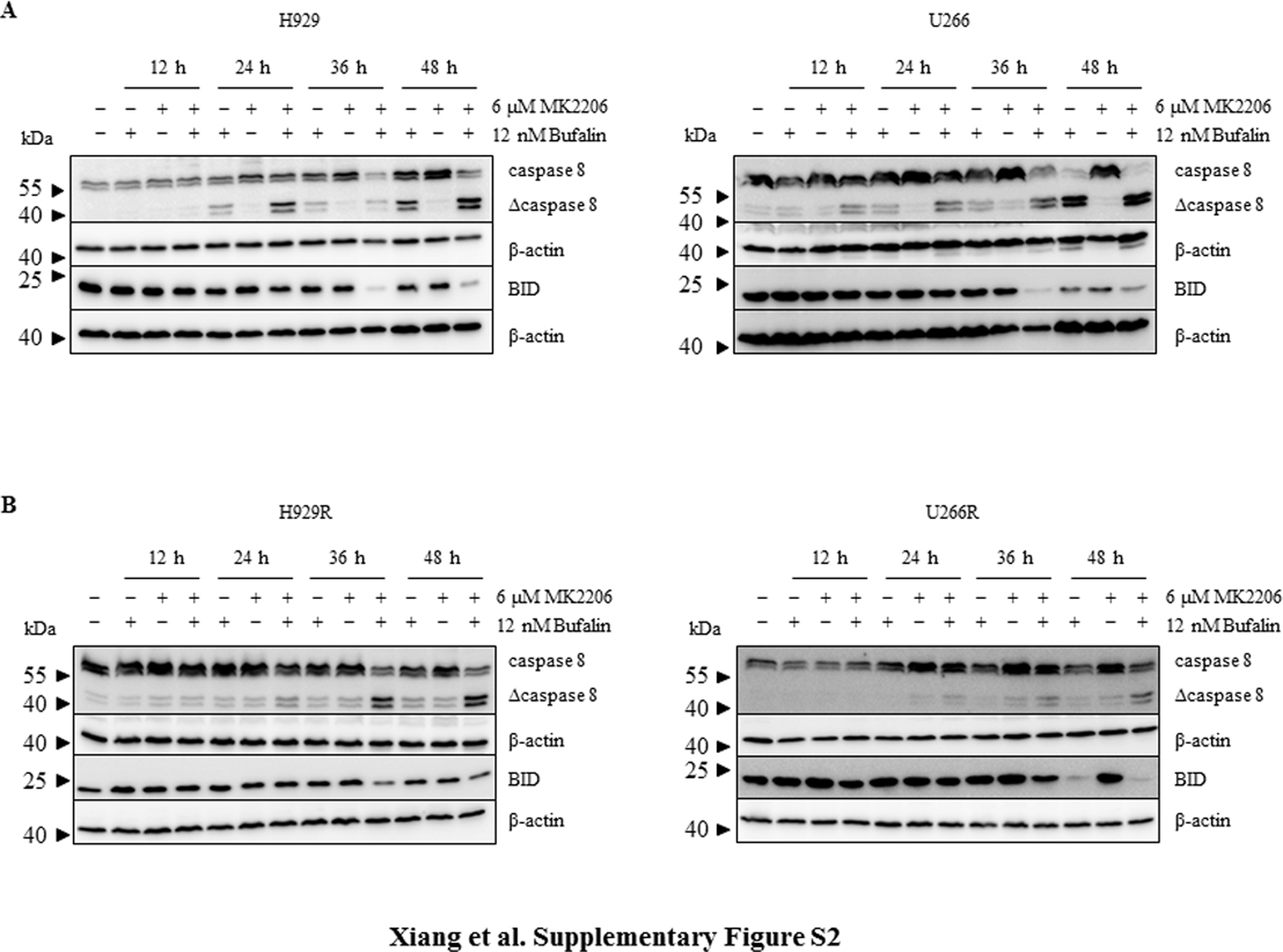

Supplement: Supplementary Figure S2 [file cddis2017188x2.tif]

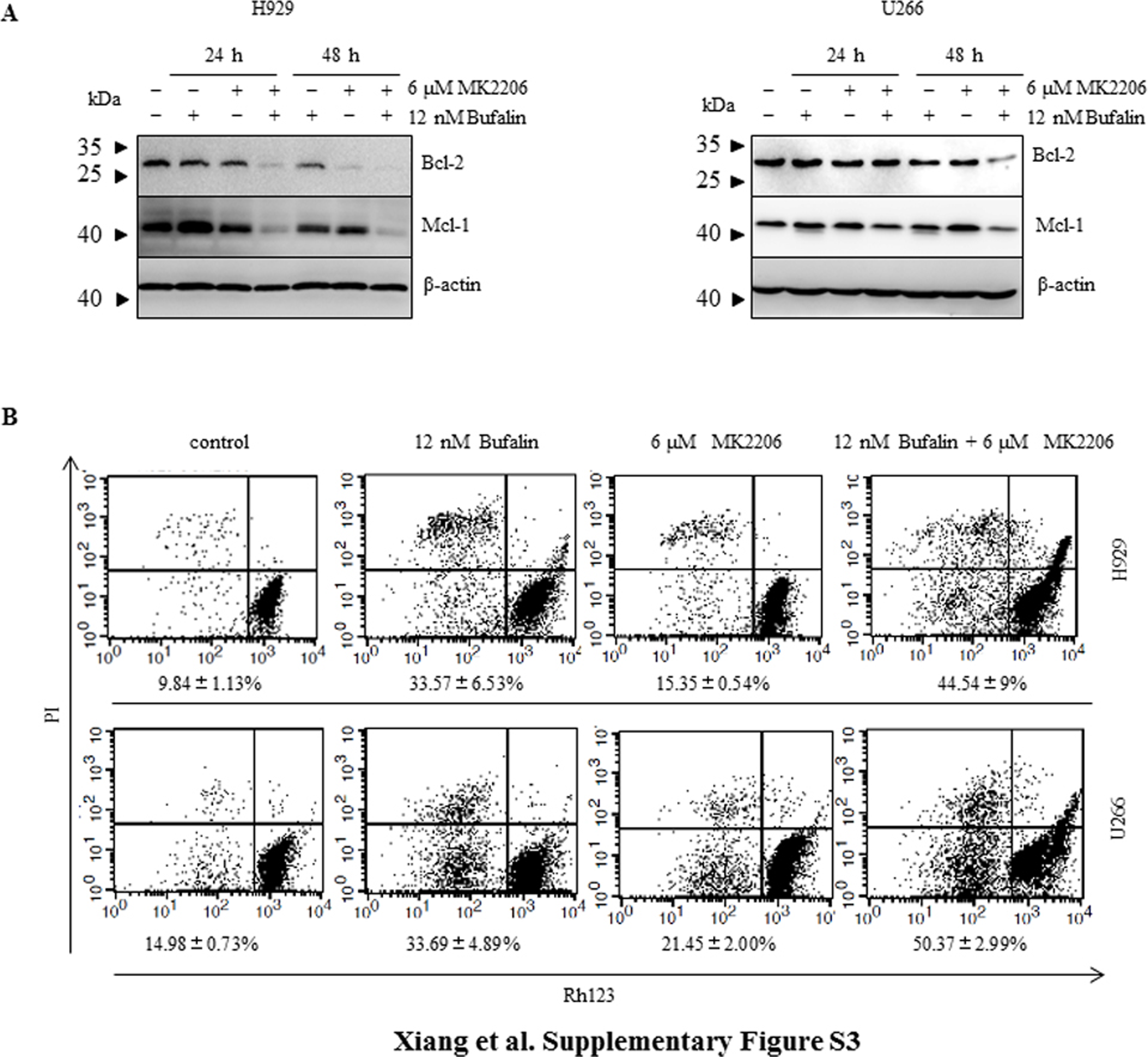

Supplement: Supplementary Figure S3 [file cddis2017188x3.tif]

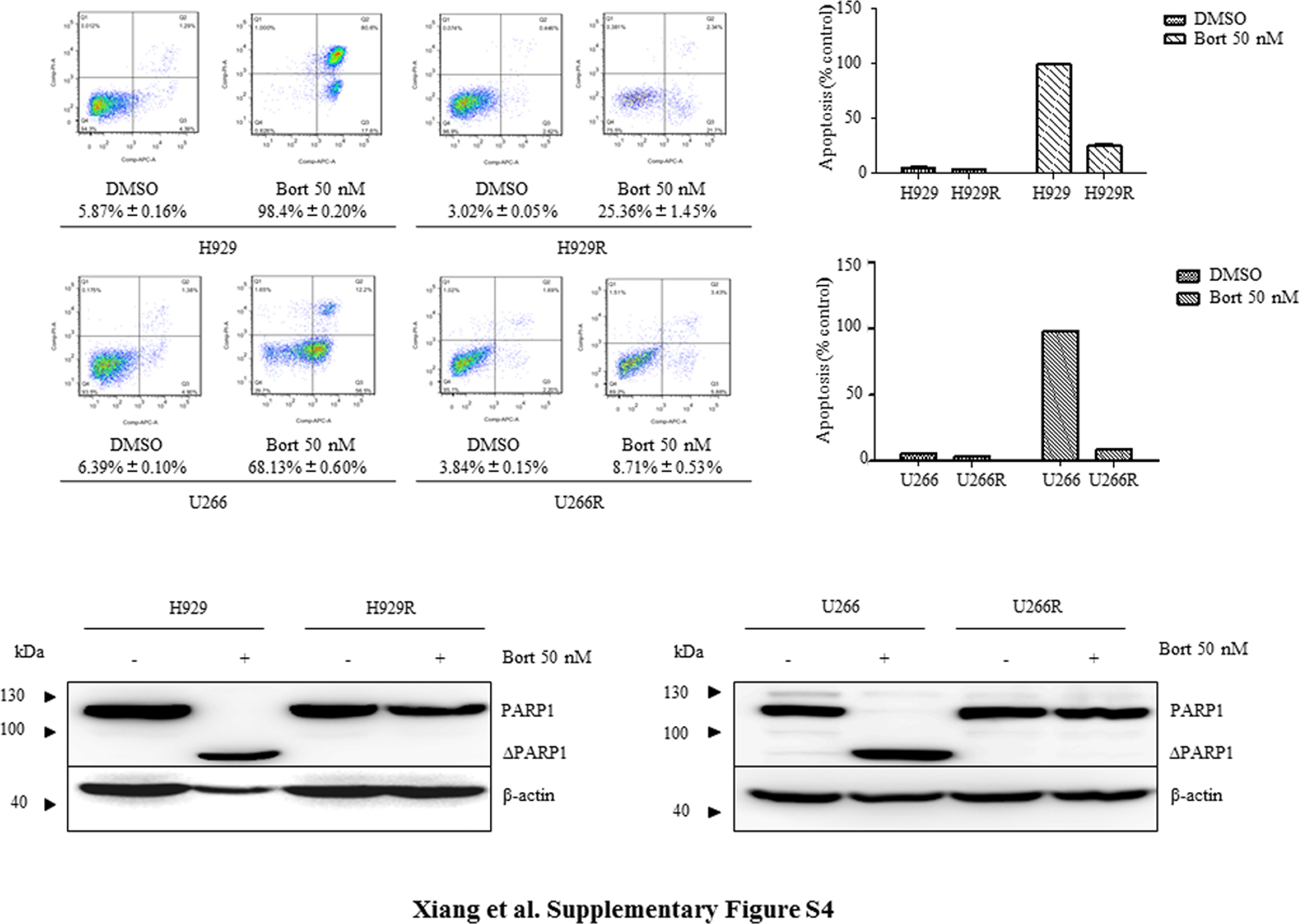

Supplement: Supplementary Figure S4 [file cddis2017188x4.tif]
