# Supplementary material for: MK2206 enhances the cytocidal effects of bufalin in multiple myeloma by inhibiting the AKT/mTOR pathway
Source: Cell Death Dis. 2017 May 11;8(5):e2776–. doi: 10.1038/cddis.2017.188 (PMC5520709; doi:10.1038/cddis.2017.188)
Supplement: Supplementary Information [file cddis2017188x5.docx]

**MK2206 enhances the cytocidal effects of bufalin in Multiple Myeloma by inhibiting the AKT/mTOR pathway**

Ru-Fang Xiang^1#^, Yan Wang^1#^, Nan Zhang^1^, Wen-Bin Xu^1^, Yang Cao^3^, Jia Tong^1^,

Jun-min Li^1*^, Ying-Li Wu^2*^, Hua Yan^1*^

^1^Department of Hematology, Rui-Jin Hospital, Shanghai Jiao-Tong University School of Medicine, Shanghai, 200025, China;

^2^Hongqiao International Institute of Medicine, Shanghai Tongren Hospital / Faculty of Basic Medicine, Chemical Biology Division of Shanghai Universities E-Institutes, Key Laboratory of Cell Differentiation and Apoptosis of the Chinese Ministry of Education, Shanghai Jiao Tong University School of Medicine, Shanghai 200025, China

^3^Department of Hematology, The Third Affiliated Hospital of Suzhou University, The First People’s Hospital of Changzhou, Changzhou, Jiangsu Province, 213003, China

^#^ Authors equally contributed this work

^*^Corresponding authors

**Figure S1. Bufalin inhibits MM cell growth in the absence and/or presence of MK2206. (A-D)** The four MM cell lines namely H929, U266, 8226 and LP-1 MM cells were treated with bufalin (1.5, 3, 6, 12, 24 nM) for 48 h in the absence and/or presence of the indicated concentrations of MK2206 (0.75, 1.5, 3, 6, 12 μM). Cell viability was measured by the CCK8 assay and the combination index was calculated by the software CompuSyn. Each bar represented the mean ± SE (standard error) of triplicate experiments (*, P < 0.05; **, P < 0.01).

**Figure S2.** **(A, B)** H929 and U266 cells were incubated with 12 nM of bufalin in the absence and/or presence of 6 μM of MK2206 for 12, 24, 36 and 48 h and protein lysates were subjected to immunoblot analysis using antibodies specific against caspase-8, BID and/or β-actin. β-actin was used as a loading control. **(C, D)** H929R and U266R cells were treated with 24 nM of bufalin in the absence and/or presence of 12 μM MK2206 for 12, 24, 36 and 48 h and protein lysates were subjected to immunoblot analysis using antibodies specific against caspase-8, BID and/or β-actin. β-actin was used as a loading control. Experiments were performed in triplicate.

**Figure S3. The apoptosis induced by combination of bufalin and MK2206 was partially related to the mitochondrial pathway. (A)** H929 and U266 cells were treated with 12 nM of bufalin in the absence and/or presence of 6 μM of MK2206 for 24 and 48 h and the levels of Bcl-2 and Mcl-1 were examined by Western blot analysis. β-actin was used as a loading control. **(B)** H929 and U266 cells were treated with 12 nM of bufalin in the absence and/or presence of 6 μM of MK2206 for 48 h, and mitochondrial transmembrane potential was detected by Rh123/PI double staining. Experiments were performed in triplicate.

**Figure S4.** Bortezomib-sensitive H929 and U266 cells and bortezomib-resistant H929R and U266R cells were treated with 50 nM bortezomib for 48 h and subjected to flow cytometry in order to measure apoptotic rates. The cells were harvested flow cytometry was conducted as shown in histograms. Cell lysates were subjected to immunoblot analysis in order to detect the cleavage of the apoptosis-related proteins PARP.
